# Meditation-Induced Near-Death Experiences: a 3-Year Longitudinal Study

**DOI:** 10.1007/s12671-018-0922-3

**Published:** 2018-03-12

**Authors:** William Van Gordon, Edo Shonin, Thomas J. Dunn, David Sheffield, Javier Garcia-Campayo, Mark D. Griffiths

**Affiliations:** 1 Centre for Psychological Research, University of Derby, Kedleston Road, Derby, Derbyshire DE22 1GB UK; 2 Awake to Wisdom Centre for Meditation and Mindfulness Research, Ragusa, Italy; 3 Psychology Division, Bishop Grosseteste University, Lincoln, UK; 4 Miguel Servet University Hospital, University of Zaragoza, Zaragoza, Spain; 5 Psychology Department, Nottingham Trent University, Nottinghamshire, UK

**Keywords:** Meditation-induced near-death experience, Near-death experience, Meditation, Death, Buddhism

## Abstract

Near-death experiences (NDEs) are life transformational events that are increasingly being subjected to empirical research. However, to date, no study has investigated the phenomenon of a meditation-induced near-death experience (MI-NDE) that is referred to in ancient Buddhist texts. Given that some advanced Buddhist meditators can induce NDEs at a pre-planned point in time, the MI-NDE may make NDEs more empirically accessible and thus advance understanding into the psychology of death-related processes. The present study recruited 12 advanced Buddhist meditators and compared the MI-NDE against two other meditation practices (i.e. that acted as control conditions) in the same participant group. Changes in the content and profundity of the MI-NDE were assessed longitudinally over a 3-year period. Findings demonstrated that compared to the control conditions, the MI-NDE prompted significantly greater pre-post increases in NDE profundity, mystical experiences and non-attachment. Furthermore, participants demonstrated significant increases in NDE profundity across the 3-year study period. Findings from an embedded qualitative analysis (using grounded theory) demonstrated that participants (i) were consciously aware of experiencing NDEs, (ii) retained volitional control over the content and duration of NDEs and (iii) elicited a rich array of non-worldly encounters and spiritual experiences. In addition to providing corroborating evidence in terms of the content of a “regular” (i.e. non-meditation-induced) NDE, novel NDE features identified in the present study indicate that there exist unexplored and/or poorly understood dimensions to NDEs. Furthermore, the study indicates that it would be feasible—including ethically feasible—for future research to recruit advanced meditators in order to assess real-time changes in neurological activity during NDEs.

## Introduction

A near-death experience (NDE) is a reported memory of a pattern of experiences that can occur when a person is close to dying (e.g. life-threatening situations, asphyxia, near-drowning, stroke, etc.), when they believe they are close to dying (e.g. shock due to loss of blood) and in the period between clinical death and resuscitation (e.g. due to cardiac arrest) (van Lommel 2011). NDEs have also been reported as occurring in non-life-threatening situations such as during grief and anxiety, epilepsy, syncope and Cotard’s syndrome (Charland-Verville et al. 2014). NDEs are often transformational, prompting (for example) enhanced intuitive sensibility; changes in life insight; greater understanding of self; and changes in spiritual and/or religious beliefs (Khanna and Greyson 2014; Schwaninger et al. 2002). Approximately 4% of adults in Western countries have experienced at least one NDE, although prevalence estimates should be treated with caution because the primary means of identifying NDEs is via self-report (e.g. individuals may be reluctant to share their experience following an NDE) (van Lommel 2011).

Although individual, cultural and religious factors influence the vocabulary individuals use to describe and interpret their NDEs, consensual scientific opinion appears to be that there is little variation in the components of NDEs (van Lommel et al. 2001). More specifically, NDEs are understood to involve one or a combination of the following features: (i) an out-of-body experience (OBE), (ii) seeing or moving through a tunnel, (iii) communicating with a being of light, (iv) observing a celestial landscape, (v) meeting with deceased persons, (vi) a life review, (vii) loss of sense of time and space and/or (viii) a conscious return to the body (Schwaninger et al. 2002; van Lommel 2011).

The typical format of NDE studies in human participants is that it follows either a retrospective (i.e. asking participants about their experience of a prior NDE) or prospective (i.e. exploring how participants’ life and perspective changes following NDEs) design. The principal reason for this is that NDEs typically occur unexpectedly, and concurrently assessing brain activity whilst an individual is experiencing an NDE would give rise to numerous logistical as well as ethical challenges. However, there does exist an electroencephalogram (EEG) study monitoring brain activity in rats following clinical death induced by euthanasia (Borjigin et al. 2013). In this study, EEG recordings identified four distinct stages of brain death: (i) cardiac arrest reflecting the time (~ 4 s) between the last regular heartbeat and the loss of an oxygenated blood pulse (i.e. clinical death; stage 1), (ii) a burst in low-frequency brain waves (~ 6 s; stage 2), (iii) increase in high-frequency gamma activity (~ 20 s; stage 3) and (iv) cessation of meaningful brain activity (stage 4). According to the study authors, the high-frequency neurophysiological activity in the near-death state (i.e. stage 3) exceeded levels found during the conscious waking state and may be similar to the highly lucid mental experiences reported by human near-death survivors.

Clearly, the extent to which findings from the aforementioned study using rats can be generalised to human participants remains highly questionable. However, in the absence of research examining human brain activity during an NDE episode, studies using mammals provide potentially useful information to guide future empirical enquiry. Another area of research that may help inform understanding of NDEs in human participants is studies assessing the neural correlates and wider participant experiences of meditation. For example, a sense of separation from the body, alterations in time and space perception and encountering mystical beings are experiences that have all been reported during meditation (Kundi 2013; Travis 2014). Furthermore, increased gamma-band activity (i.e. similar to outcomes of the aforementioned rat study) has been observed in advanced Buddhist meditation practitioners both during and following meditation (Lutz et al. 2004). Indeed, when meditators were asked to mentally visualise and emotionally connect with encountering a “being of light” typical of an NDE, they displayed greater gamma activity and other neuro-electric changes in brain regions associated with positive emotions, imagery, attention and spiritual experiences (Beauregard et al. 2009).

The practice of using meditation to derive a better understanding of death is longstanding. This is particularly the case in Buddhism where ancient texts exist that describe meditation practices specifically intended to help spiritual practitioners prepare for, or gain insight into, the processes of dying and death. An example that has gained some popularity in the West is the eighth-century *Tibetan Book of the Dead* (Padmasambhava, 8^th^ century/1994) along with more recent adaptations such as the *Tibetan Book of Living and Dying* (Sogyal 1998). A related (but less well-known) text by the same eighth-century author is the *Profound Dharma of Natural Liberation Through Contemplating the Peaceful and Wrathful: Stage of Completion Instructions on the Six Bardos* (Padmasambhava, 8^th^ century/1998). These texts outline specific phases that the consciousness is purported to progress through during and following death.

Another notable Tibetan Buddhist text is *Delok: Journey to the Realms Beyond Death* (Drolma 1995) that recounts the author’s experience of being a *delog*. According to Tibetan Buddhism, *delogs* are individuals that experience (sometimes lasting hours or even days) NDEs and subsequently share their experiences as a means of assisting other spiritual practitioners (Baily 2001). In line with the content of each of the aforementioned Tibetan Buddhist works, the Dalai Lama (2006) asserted that at the moment of death, a state of consciousness briefly manifests in all human beings which has the properties of being subtle, spontaneous, and without self-grasping. The Dalai Lama (2006) also asserted that experienced meditators can deliberately induce this state during meditation such that when it naturally occurs at death, they can recognise and sustain it.

Additional relevant texts that exert a key role in Theravada Buddhism are the *satipaṭṭhāna sutta*, *mahasatipaṭṭhāna sutta* and *kāyagatāsati sutta.* Each of these discourses contains the *nine charnel ground contemplations* that are intended to help the meditation practitioner move beyond being attached to their body and gain insight into the processes of decomposition and dissolution that occur during and following death (Shonin and Van Gordon 2014a). Other Theravada Buddhist texts such as the *assalāyana sutta* acknowledge the existence of the *gambhava* which can be interpreted as the “external consciousness” or “aspiring consciousness” that manifests after death (Anālayo 2008). Furthermore, accounts of the Buddha guiding his disciples to experience the hells and other realms of existence could also arguably be interpreted as a form of NDE.

There are several documented explanations as to why some Buddhist practitioners engage in meditation techniques intended to foster familiarisation with death. A key reason is to overcome attachment to the body and to the idea that the “self” exists as an enduring, independent entity (Sogyal 1998). More specifically, Buddhism asserts that suffering arises as a result of an individual’s “attachment” to both themselves and external phenomena (e.g. wealth, people, reputation, etc.; Feliu-Soler et al. 2016). The Buddhist notion of attachment has been defined as “the over-allocation of cognitive and emotional resources towards a particular object, construct, or idea to the extent that the object is assigned an attractive quality that is unrealistic and that exceeds its intrinsic worth” (Shonin et al. 2014, p. 126). Consequently, in the Buddhist meditation literature, reducing attachment (or augmenting non-attachment) is deemed to be an important feature of the path to psycho-spiritual wellbeing (Van Gordon et al. 2017). A second reason for increasing familiarity with death is that given it is a process that affects every living being, it is arguably wise to prepare for and/or try to understand it (Shonin and Van Gordon 2014a). Furthermore, some Buddhist practitioners use meditation to induce so-called mystical experiences (i.e. such as those that comprise NDEs) in order to understand that such experiences are, in fact, not mystical but are “normal” dimensions of the mind and reality that can be accessed through meditation (Shonin et al. 2014).

Thus, in Tibetan Buddhism and to a lesser extent in other Buddhist traditions such as Theravada Buddhism, there exists the view that some advanced meditators can use meditation in order to gain insight into the state of consciousness that manifests after death. However, to date, no study has empirically investigated the phenomenon of the meditation-induced near-death experience (MI-NDE). Consequently, the following questions remain unanswered: (i) Are the MI-NDEs referred to in ancient Buddhist texts still experienced by advanced meditation practitioners today (i.e. does the MI-NDE exist as an empirically investigable phenomenon)?, (ii) What are the defining features of MI-NDEs and do they meet the criteria of a conventional NDE?, (iii) Is there a relationship between the duration and profundity of an MI-NDE?, (iv) Does familiarisation with the MI-NDE over time lead to more lucid and/or profound experiences of the after-death state? and (v) How can MI-NDEs advance scientific understanding of the NDE phenomenon and of the dying process more generally? The present study sought to address these questions by quantitatively assessing changes in the content and profundity of MI-NDEs elicited by advanced Buddhist meditators over a 3-year period (study 1) and employing qualitative analytical techniques (study 2) to identify common themes in terms of meditators’ experiences of the MI-NDE.

## Study 1

### Method

#### Participants

An often overlooked limitation of studies involving advanced Buddhist meditators is that there exist no reliable screening tools or easily observable criteria for establishing whether a meditation practitioner is at an advanced stage of practice. More specifically, being “advanced” in terms of meditation experience is not simply a function of years spent in training, self-professed proficiency and/or titles awarded by a given Buddhist tradition. Indeed, it is not uncommon for advanced Buddhist meditators to conceal their meditative abilities and insights (Shonin and Van Gordon 2015). Therefore, based on the professional opinion of the first and second authors (that have over 40 years’ collective experience as Buddhist meditation teachers), purposive sampling was preferred over an open call for participants (i.e. as the latter approach would likely exert unrealistic demands on the research team’s resources and result in a large proportion of participants not meeting the screening criteria).

Consequently, participant recruitment occurred via (i) contacting individuals known by two members of the research team to be at an advanced stage of meditation practice, (ii) contacting individuals known by two members of the research team to have engaged in the MI-NDE practice and (iii) providing information about the study to Buddhist teachers (i.e. known for being astute in terms of appraising an individual’s meditative competency) in lay and monastic practicing Buddhist communities and asking them to forward the contact details of the research team to appropriate individuals. Participant recruitment spanned all three of the major Buddhist traditions (i.e. Theravada, Mahayana and Vajrayana) and was global in terms of geographical scope. Given that some advanced Buddhist meditation practitioners have limited access to communication media and/or choose to live in isolation for long periods of time, the recruitment window remained open for 12 months.

To be included in the study, participants had to score 7 or more on the *Near-Death Experience Scale* (Greyson 1983) in respect of their most recent MI-NDE. Furthermore, participants had to be (i) aged 18 years or older, (ii) able to speak and read English, (iii) planning to undergo the MI-NDE practice at a frequency of at least once per year for the next 3 years, (iv) not currently diagnosed with a psychotic disorder and (v) not currently using psychopharmacological or recreational drugs.

Participant demographic characteristics are shown in Table 1. A total of 38 Buddhist meditation practitioners expressed an interest in the study and 26 of these were screened out on the grounds of ineligibility. The main reasons for exclusion were they (i) did not score 7 or more on the NDE Scale (11 participants), (ii) could not speak and/or read English (8 participants) and (iii) did not plan to engage in the MI-NDE practice at a frequency of at least once per year for the next 3 years (7 participants). Of the remaining 12 eligible participants, 83.3% were male and two-thirds (66.7%) were Buddhist monastics (i.e. with the remainder being lay Buddhist practitioners). The average age of participants was 54.17 years (SD = 8.53) and the average meditation experience was 28.08 years (SD = 10.83). On average, participants engaged in the MI-NDE practice three times per year (SD = 1.35). All 12 eligible participants completed semi-structured interviews (SSIs) and all participants completed psychometric assessments for year 1. One participant dropped out during year 2 and a further participant during year 3 (both of these participants became uncontactable and did not provide a reason for discontinuing).

**Table 1:** Participant demographic information

Participant number	Sex (M/F)	Age at year 1 (years)	Meditation experience (years)	Buddhist affiliation	Monastic/lay	Nationality	Ethnicity	Lost to follow-up	No. of MI-NDEs per year (mean)
1	M	62	35	Theravadan	M	Sri Lankan	Asian	n	3
2	M	43	25	Theravadan	M	Sri Lankan	Asian	n	2
3	M	56	18	Vajrayana (Tibetan)	L	British	White	n	4
4	F	54	39	Theravadan/Mahayana	M	Thai	Asian	n	5
5	M	46	18	Mahayana/Vajrayana (Tibetan)	L	Nepalese	Asian	y	1
6	M	63	25	Vajrayana (Japanese)	M	Japanese	Asian	n	3
7	M	39	15	Thera/Maha/Vajra (Tibetan)	M	British	White	n	5
8	M	48	18	Mahayana/Vajrayana (Tibetan)	M	Indian	Asian	n	4
9	M	59	51	Thera/Maha/Vajra (Tibetan)	L	Italian	White	n	2
10	M	66	36	Vajrayana (Tibetan)	L	Tibetan	Asian	y	3
11	F	54	24	Theravadan/Mahayana	M	Sri Lankan	Asian	n	3
12	M	60	33	Theravadan	M	Sri Lankan	Asian	n	1

#### Procedure

Participants were required to complete a battery of psychometric scales in respect of their first MI-NDE of each calendar year for a period of 3 years (participants were only required to complete scales relating to mysticism and non-attachment in respect of the first MI-NDE they experienced in year 1). Participants were required to complete the scale within 24 h of concluding a given MI-NDE practice and then once again within 24 h of concluding the practice (and prior to engaging in any other form of formal meditation practice).

##### Control Condition

Participants were also requested to complete psychometric tests in year 1 in respect of a (i) standard meditation practice that did not induce the NDE or involve any form of contemplation on death or death-related processes and (ii) standard meditation practice that involved reflecting on death and/or death-related processes but that did not induce the NDE. A meditation was deemed to be standard if it (i) took the form of a formal seated meditation session, (ii) was at least 45 min in duration and (iii) primarily involved cultivating concentration and mindfulness.

Due to the very small number of advanced meditators that can induce an MI-NDE, recruiting a control group with comparable meditative capabilities was a logistical challenge. Indeed, even though recruitment was global in scope and spanned a 12-month period, only 12 individuals were identified whom could induce an MI-NDE according to the pre-defined screening criteria (i.e. and whom consented to participate in the study). Consequently, a within-participant control condition was deemed to be the most effective means of assessing the effects of the MI-NDE against other forms of advanced meditation practiced by individuals with equivalent meditative capabilities.

##### Ethics

Ethical approval was provided by the researchers’ university research ethics committee. As part of the informed consent procedure, participants were required to confirm that they understood that the scope of the study was limited to asking them to share information about their engagement in an MI-NDE practice that they would in any event be undertaking as part of their spiritual/religious training or beliefs. Furthermore, participants were required to confirm that they understood that at no point would they be required or requested to implement the MI-NDE procedure solely for the purposes of providing data for the present study. Participation in the study was on a voluntary basis and participants did not receive any incentive for their participation.

#### Measures

Participants were requested to complete the following assessment tools:

*Near-Death Experience Scale* (NDE Scale; Greyson 1983): The 16-item NDE Scale assesses cognitive (e.g. accelerated thinking), affective (e.g. feelings of joy, peace and harmony with the universe) and paranormal (e.g. an OBE, seeing scenes from the future) components of an NDE. Participants rate each item from 0 to 2 (0 = not present, 1 = mildly or ambiguously present and 2 = definitely present) and total scores range from 0 to 32. The mean score for individuals that have experienced an NDE is 15, and a score of 7 (i.e. one standard deviation below the mean) is considered to be the cut-off for an NDE (Greyson 1983). The NDE Scale has been shown to differentiate NDEs from other close encounters with death (Greyson 1990) and to be a reliable tool irrespective of gender, age, intensity of experience and/or time elapsed since the NDE (Lange et al. 2004).

*The Mysticism Scale* (Hood 1975): The 32-item Mysticism Scale is the most widely used psychometric instrument for assessing mystical experience. It assesses mystical experience across the eight domains of positive affect, sacredness, noetic quality (sensation of the experience as a source of valid direct knowledge), unity in diversity, inner subjectivity, loss of selfhood, timelessness and spacelessness, and ineffability. Each domain includes four items of which two are positively worded and two negatively worded. Responses are converted to a five-point Likert scale (1 = low, 5 = high) that corresponds to the extent that participants’ experiences accord with each of the 32 statements. Total scores range from 32 to 160 and a single item score of 4 or more is typically deemed to constitute a mystical experience for the item in question. The scale has demonstrated good cross-cultural reliability (Hood et al. 2001).

*Non-Attachment Scale* (NAS; Sahdra et al. 2010): The 30-item NAS is based on Buddhist philosophy and assesses the degree to which an individual becomes attached to their experiences on the psychological, social and environmental plane. The NAS also assesses the degree to which a person is “attached to themselves” because according to Buddhist theory, attachment to psychological or environmental phenomena arises due to a firm sense of selfhood (Shonin et al. 2016). The NAS is constructed upon the Buddhist notion that the self does not inherently exist and that attachment to self and environment thus constitutes a maladaptive condition (see Shonin et al. 2014 for a discussion of the differences between Buddhist and Western psychological conceptualisations of attachment). The NAS is scored on a six-point Likert scale (from 1 = *disagree strongly* to 6 = *agree strongly*) and features items such as “I can admit my shortcomings without blame or embarrassment” and “When pleasant experiences end, I am fine moving on to what comes next”. The maximum score is 180 and higher scores reflect lower levels of attachment (or higher levels of non-attachment).

*Duration of the MI-NDE*: In respect of their first MI-NDE of each calendar year for a period of three years, participants were requested to record the duration of the MI-NDE to the nearest minute (i.e. as measured by a wrist watch or wall clock).

#### Data Analysis

A significance level of *p* < 0.05 and two-tailed tests were employed throughout. Where appropriate, alpha levels were increased to control for family-wise error rate (probability of a type 1 error). All consequent *p* values are reported in light of any adjustments. Owing to the sample size, non-parametric methods were employed to test for significant relationships (Spearman’s Rho) and differences between independent groups (Mann-Whitney *U*). Multi-level modelling using rank transformations was employed to test for differences across related measurements (e.g. time interval). This method provides a more robust test with greater statistical power than the Friedman test and allows for an unbalanced design (Baguley 2012; Zimmerman and Zumbo 1993).

### Results

#### Analysis of Outcome Measures

**Fig. 1:**
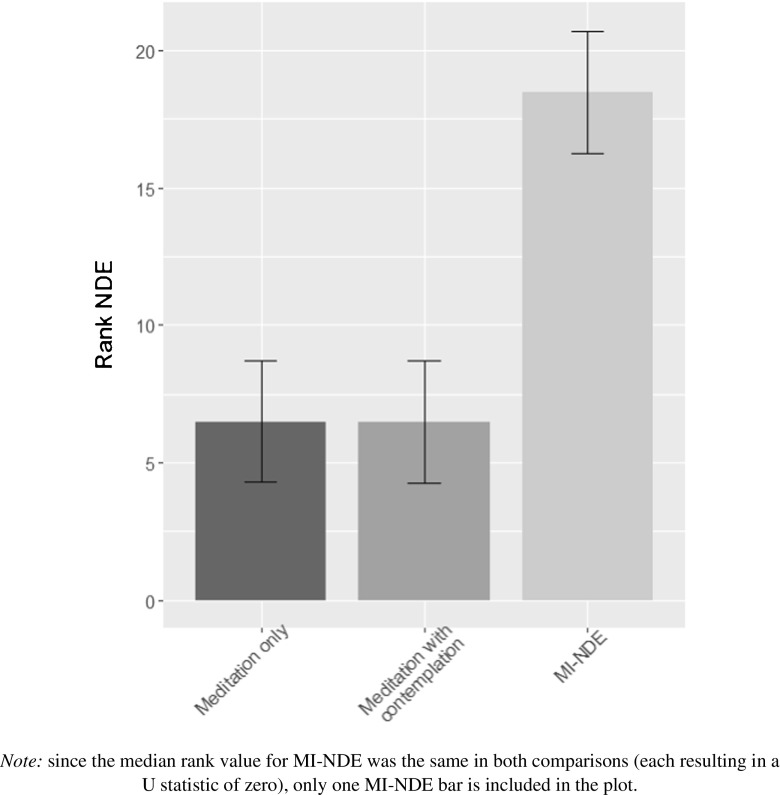
Median rank NDE scores for the experimental and control groups with 95% confidence intervals. *Note:* Since the median rank value for MI-NDE was the same in both comparisons (each resulting in a *U* statistic of 0), only one MI-NDE bar is included in the plot

Results showed no significant relationship between duration of MI-NDE and profundity (as assessed by the NDE Scale) for each year of assessment (year 1: Rho [12] = − 0.07, *p* = 0.82; year 2: Rho [11] = 0.35, *p* = 0.29; year 3: Rho [10] = 0.36, *p* = 0.29). Mann-Whitney *U* tests were employed to test for differences in NDE Scale scores between the MI-NDE condition and each of the control conditions (i.e. standard meditation and standard meditation with death contemplation). Results showed significant differences in NDE Scale scores between MI-NDE and standard meditation (*U* = 0, *p* < 0.001) and between MI-NDE and the standard meditation with death contemplation (*U* = 0, *p* < 0.001) (see Fig. 1).

**Fig. 2:**
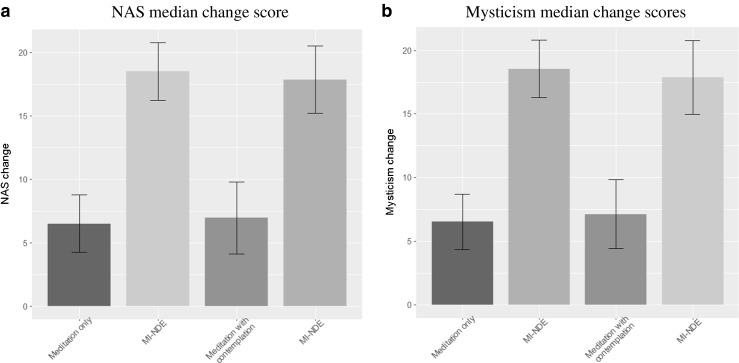
Median rank for NAS and mysticism change scores (pre-post meditation) with 95% confidence intervals. **a** NAS median change score. **b** Mysticism median change scores

Mann-Whitney *U* tests showed significant differences in mysticism change score (pre-post meditation) between the MI-NDE condition and both control conditions (standard meditation [*U* = 0.5, *p* < 0.001] and standard meditation with death contemplation [*U* = 0, *p* < 0.001]). Further Mann-Whitney *U* tests showed significant differences in NAS change score (pre-post meditation) between the MI-NDE condition and both control conditions (standard meditation [*U* = 0, *p* < 0.001] and standard meditation with death contemplation [*U* = 0, *p* < 0.001]). Figure 2 displays the NAS (Fig. 2a) and Mysticism Scale (Fig. 2b) change scores across groups (MI-NDE vs. standard meditation, MI-NDE vs. standard meditation with death contemplation).

**Fig. 3:**
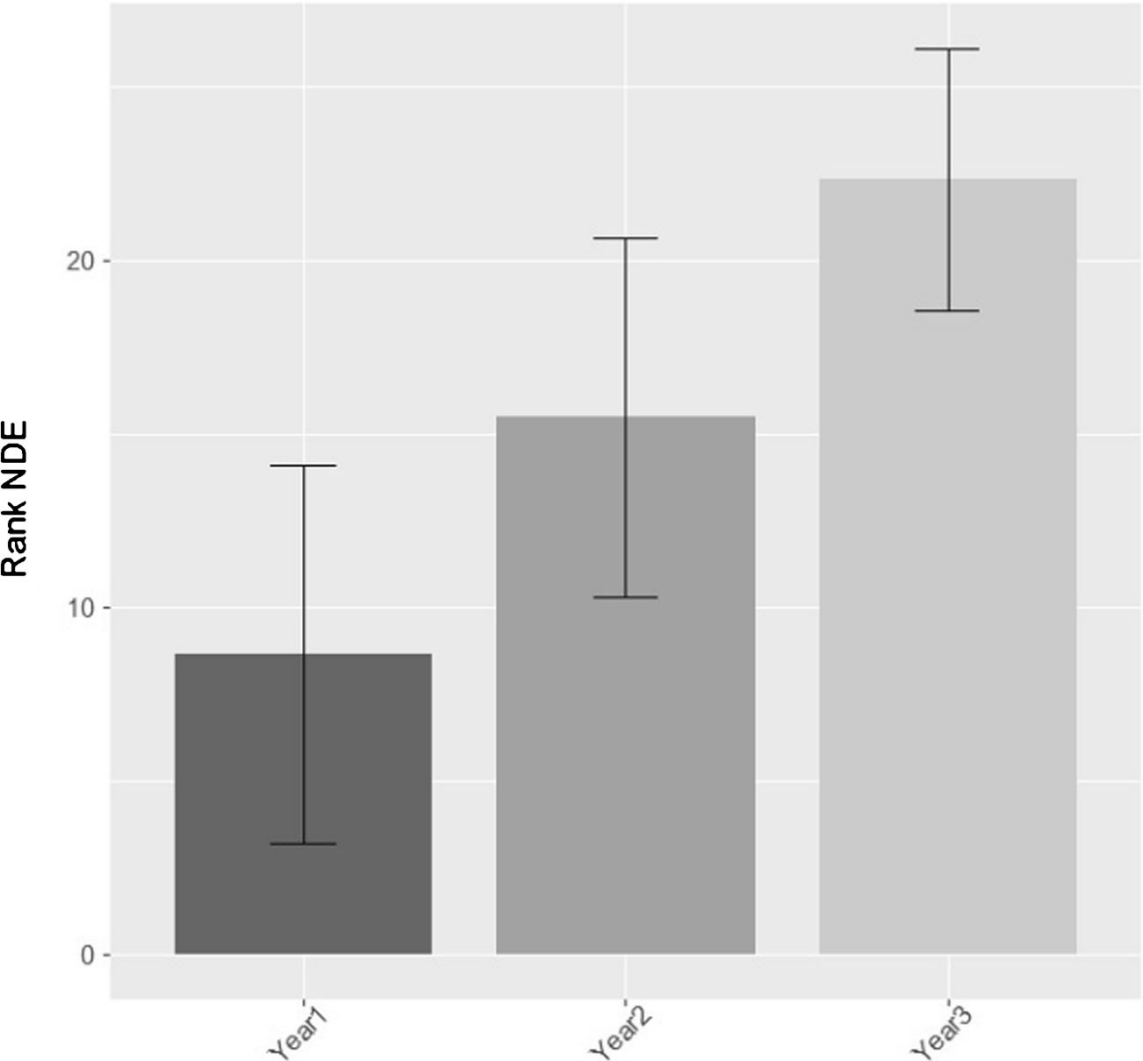
Mean rank NDE score across years with 95% confidence intervals

A rank transformation of NDE followed by a multi-level model (equivalent to a one-way repeated ANOVA) was performed to test for differences in NDE score across years (see Fig. 3). The multi-level model treated year as a fixed effect with participant as a random effect and showed a significant change in NDE score across year (*F* value [22] = 30.59, *p* < 0.001).

#### Demographic Analysis

A series of Spearman’s correlations were conducted to examine relationships between demographic characteristics and profundity of NDE (at years 1, 2 and 3). Results showed a statistically significant relationship between participants’ meditation experience (as measured in years) and NDE profundity. This was significant at year 2 (Rho [11] = 0.80, *p* < 0.01) and year 3 (Rho [10] = 0.73, *p* < 0.05) and close to significance at year 1 (Rho [12] = 0.53, *p* = 0.07), suggesting profundity of NDE increases commensurate with years of meditation experience. There were no significant relationships between age or average number of NDEs experienced per year and NDE profundity.

## Study 2

### Method

#### Participants

Each participant of study 1 underwent one SSI that corresponded to their first MI-NDE of year 1 (or of year 2 or 3 in the event the participant was unavailable to complete the SSI in year 1).

#### Procedure

Participants were requested to contact the research team as soon as possible after having completed an MI-NDE practice. An SSI was then conducted in which participants were asked a series of questions about their experience. To reduce recall bias due to the time-lag between MI-NDE and interview, interviews using internet-based video conferencing media were permitted. The SSI questions focused on (i) *content* (e.g. Did you visit non-worldly realms?, Did you possess a corporeal form?, Were you able to move unimpeded through physical objects?, Did you experience a review of this or previous lifetimes?, Are there any other key features of the MI-NDE that we have not discussed?, etc.), (ii) *awareness* (e.g. Were you aware of your physical worldly body whilst experiencing the MI-NDE?, Could you hear sounds in the vicinity of your physical worldly body?, Were you fully conscious of the fact that you were experiencing an NDE?, etc.), (iii) *volitional control* (e.g. Were you able to influence which particular non-worldly realm you visited?, Did you have control over how long you stayed in such a realm?, Did you choose to terminate the MI-NDE or did it terminate of its own accord?, etc.) and (iv) *meaning* (e.g. Why do you choose to undergo the MI-NDE practice?, What prompted you to undergo the practice on this particular occasion?, How did the practice help in terms of your spiritual development?, etc.). Open questioning was employed in order to encourage participants to freely express themselves, and Socratic questioning was used to elicit further clarification as required. The SSIs were audio recorded and then transcribed verbatim.

#### Data Analysis

Grounded theory (Glaser and Strauss 1967) uses a systematic set of procedures to formulate an inductively derived theory concerning the phenomenon under investigation (Mason and Hargreaves 2001). Transcripts were read several times and coded to identify and isolate components, experiences and significant events. In vivo codes (i.e. drawn directly from the participants’ accounts) were employed as much as possible in order to capture participants’ experiences in their own words (Strauss 1987). Categories, concepts and patterns of meaning were subsequently identified, and transcripts were assessed for divergence and convergence. The researchers continuously interacted with the data, identifying the relationships between concepts and asking questions to generate theory (Mackenzie et al. 2007; Strauss and Corbin 1990). From the initial formulation of codes until the emergence of master and subordinate themes, “bracketing” (Creswell 2007) was employed to minimise the influence of the researchers’ biases and assumptions relating to the MI-NDE phenomenon. Grounded theory requires sufficient raw evidence to establish the validity of the constructed theory (Henwood and Pidgeon 1993; Mason and Hargreaves 2001). Consequently, the “Results” section that follows makes moderate use of direct excerpts from participant transcripts. For the purposes of validation, the entire analytical process, from reading the raw data through to identifying themes, was repeated in iterative fashion until saturation was achieved (Van Gordon et al. 2016a). The analytical process was repeated by a second member of the research team as a form of independent audit. Additional validation techniques such as grounding in examples and requesting feedback from participants on the summary of themes were also employed (Creswell 2007).

### Results

The analysis of participants’ transcripts generated four master themes, each with a different number of subordinate themes. The final hierarchical thematic structure is shown in Table 2, and a description of the emerging master and subordinate themes (including illustrative verbatim extracts) is provided below.

**Table 2:** Summary of master and subordinate themes

Master theme	Subordinate themes
1. Phasic and multi-faceted NDE	1.1 Identification with the elements
	1.2 Altered perception of time and space
	1.3 Non-worldly encounters
	1.4 Emptiness
2. Awareness during the MI-NDE	2.1 Awareness of physical worldly body
	2.2 Awareness of NDE and non-corporeal form
3. Volitional control	N/A
4. Spiritually meaningful insights	N/A

#### Phasic and Multi-faceted NDE

This master theme related to the fact that all participants experienced a rich and multi-faceted MI-NDE that comprised discrete phases. The master theme comprised four sub-themes that are presented chronologically in terms of the order they were reported to unfold during the MI-NDE.

##### Identification with the Elements

All participants reported that the MI-NDE began with them consciously reducing the degree of connection to their physical worldly body. Participants referred to this as a process of “gradual dissolution” (participants 1, 6 and 10), “letting go of body” (participants 2, 5 and 11) or “becoming untied” (participants 3 and 8), and it corresponded to specific visions that they attributed to the sequential unbinding of five bodily elements (i.e. beginning with earth, then water, then sun/fire/heat, then air/wind and concluding with space). More specifically, participants reported that they experienced (for example) a feeling of drowning (i.e. as they lost connection with water), being stuck and/or unable to move (i.e. as they lost connection with earth), being too hot or too cold (i.e. as they lost connection with fire), breathlessness and weightlessness (i.e. as they lost connection with air) and being without a body (i.e. as they lost connection with space).

##### Altered Perception of Time and Space

All participants reported that during the next phase of the MI-NDE, they ceased to be aware of time and space. More specifically, participants explained that rather than becoming unaware of time or space, they realised that time and space are relative phenomena that ultimately do not exist. Participants’ sentiments concerning their altered perception of time and space are best captured by the following excerpt from participant 7:

When I’m not meditating, I know that time and space don’t exist. I know it and I remember it, but I don’t experience it. But the [MI-NDE] allows me to experience it directly. I am nowhere and I am everywhere. The past, present, and future merge into one. (participant 7)

Ten participants stated that as part of this timeless perspective, they were able to see scenes of the past and future. This included scenes relating to their own past and future, as well as scenes relating to this and other worlds. However, such visions were deemed by participants to be “normal” (participants 2 and 3), “no big deal” (participant 4) and “something that you experience and then let go of” (participant 11). Participants 5 and 9 did not report such an experience for the NDE in question but explained that precognitive abilities had been a feature of previous NDEs.

##### Non-worldly Encounters

All participants reported that the next phase of the MI-NDE involved encounters with non-worldly realms and beings. More specifically, participants recounted that they experienced (i) undesirable realms (that they referred to using terms such as “hell” [participants 2, 5, 6, 8 and 12], realms of “torture” [participants 1, 4 and 11], worlds where the beings “hang from ropes” [participants 3 and 7] and “hungry ghost realms” [participants 9 and 10]), (ii) other realms comprising humans and animals and (iii) realms where the inhabitants were partially or fully composed of light (that participants referred to using terms such as “heavens” [participants 1, 5, 6, 7 and 11], “godly realms” [participants 2, 4, 7 and 12] and “asura realms” [participants 3 and 9]). All participants (except participant 5) also made reference to encounters with three other categories of non-worldly beings: (i) recently deceased beings in the process of moving between worlds, (ii) demonic beings attempting to enter this or other realms and (iii) liberated beings not bound to a given worldly or non-worldly realm. In respect of this latter group comprising liberated beings, all participants (except participants 5 and 12) specifically reported they met with their spiritual teacher (whether living or deceased) who supported and guided them during (and following) the NDE.

##### Emptiness

Although participants gave rich accounts of the content of their MI-NDE, they referred to this content as being of the nature of “emptiness” (participants 3, 4, 6, 7, 8, 9 and 12), “voidness” (participants 2, 5, 9, 10 and 11) and/or “non-self” (participants 1, 2, 4, 11 and 12). The following excerpt from participant 6 best captures how participants related to the MI-NDE in this respect:

Emptiness is the way things are. Its why you don’t hold on [to the MI-NDE]. You experience something or someone [during the MI-NDE] and you’re fully involved in the experience. But you pull back and recognise that it’s like a dream. It’s dangerous not to do that. If you don’t pull back, you can get caught [in the MI-NDE]. (participant 6)

Participants 3, 6, 7, 8 and 10 went one step further and stated that not only were their MI-NDE experiences empty of inherent existence, but they were “mind-made”. Participant 8 clarified their sentiments on this point as follows:

The mind has unlimited potential. But it gets stuck in one way of seeing things … But when you die the mind kind of goes through a process of unfolding. Its potential is unlocked. If you’ve trained enough you can harness this [potential] – you can harness it before you die as well. But if you’re not trained – and most people aren’t – then [death] is a frightening experience and there’s no way of controlling it. But it’s all just a projection of the mind. (participant 8)

#### Awareness During the MI-NDE

This master theme referred to the extent to which participants were consciously aware that they were experiencing an NDE as well as the extent that they remained aware of their body (both their physical worldly body and any bodily form they assumed during the MI-NDE). The master theme comprised two sub-themes that refer to participants’ awareness of their physical world and body and non-physical (i.e. NDE) world and body, respectively.

##### Awareness of Physical Worldly Body

All participants reported that during the MI-NDE, they remained meditatively aware of their physical worldly body but described it as being a “partial” (participants 1, 6, 7 and 11) or “distant” (participants 3, 6 and 9) form of awareness. Participant 4 explained this process as follows:

You leave a small thread that you use to remain in contact [with the physical worldly body] and you use it to return to the body. (participant 4)

Participants qualified this partial awareness by explaining that if during the MI-NDE their physical worldly body became (for example) cold, was subjected to pain or suddenly encountered loud noises, they would recognise such changes. However, all participants explained that during the MI-NDE, meditative awareness was primarily focussed on the NDE itself and that allocating attentional resources to their physical worldly body could interfere with (participants 1, 3, 4, 5, 8, 11 and 12), or even cause a termination of (participants 1, 2, 4, 5, 7, 9 and 10), the MI-NDE.

##### Awareness of NDE and Non-corporeal Form

All participants reported that they retained full meditative awareness and control over the fact they were experiencing NDEs. Furthermore, participants (all except participants 1 and 5) reported that they could exercise choice in terms of whether or not they assumed a bodily form during the MI-NDE. Participants also explained that in the event they did assume a bodily form, (i) they were fully aware of it, and (ii) it possessed abilities that would defy conventional physical laws. Participants 4 and 9 explained this as follows:

During [the death process], the mind of most people wants, or needs, to take on a bodily form. It doesn’t know what to do with itself otherwise. But there is really no need to [take on a bodily form]. (participant 4)

You can assume whatever [bodily form] most suits yours or others’ needs at that given time. And there are no limitations. [The body] can move through walls, fly, and instantly [translocate]. And with practice, it can even be in two places at once. (participant 9)

#### Volitional Control

This master theme, that did not comprise any subordinate themes, related to the extent to which participants retained volitional control over the content and duration of their MI-NDE. All participants explained that to differing degrees, they could voluntarily induce and terminate the NDE, and that although they could not dictate the content of an encounter with a given non-worldly being or realm, they retained control over (i) which non-worldly realm and/or being they visited and (ii) the duration of the encounter. Five participants (2, 6, 7, 9 and 11) shared their view that individuals without extensive meditative and/or spiritual experience would not be able to retain volitional control over an NDE.

#### Spiritually Meaningful Insights

This master theme, that likewise did not comprise any subordinate themes, related to the meaning that participants assigned to the MI-NDE. Participants provided a variety of reasons for engaging in the MI-NDE practice including (i) “preparing for death” (participants 1, 2, 6, 9, 10 and 12), (ii) “preparing for life” (participants 2, 3 and 9), (iii) “letting go of body” (participants 1, 3, 4 and 11), (iv) “advancing spiritually” (participants 3, 5, 8, 10 and 12), (v) “helping [non-worldly] beings” (participants 1, 7 and 10) and (vi) “letting go [in general]” (participants 1, 3, 7 and 12). Furthermore, all participants reported that engaging in the MI-NDE gave them access to spiritual insights that helped to augment their meditative awareness both during and following the MI-NDE, and that would not be accessible during a standard meditation practice. Participants (all except 5, 9 and 12) expanded on this and stated that in addition to gleaning insight into death-related processes, the MI-NDE sometimes allowed them to discover “treasures” (participants 3, 4, 6, 8 and 10) or “gifts of wisdom” (participants 2, 3, 7 and 10) that had been “placed in their mind” (participant 3) by their spiritual teacher.

#### Theory Building

**Fig. 4:**
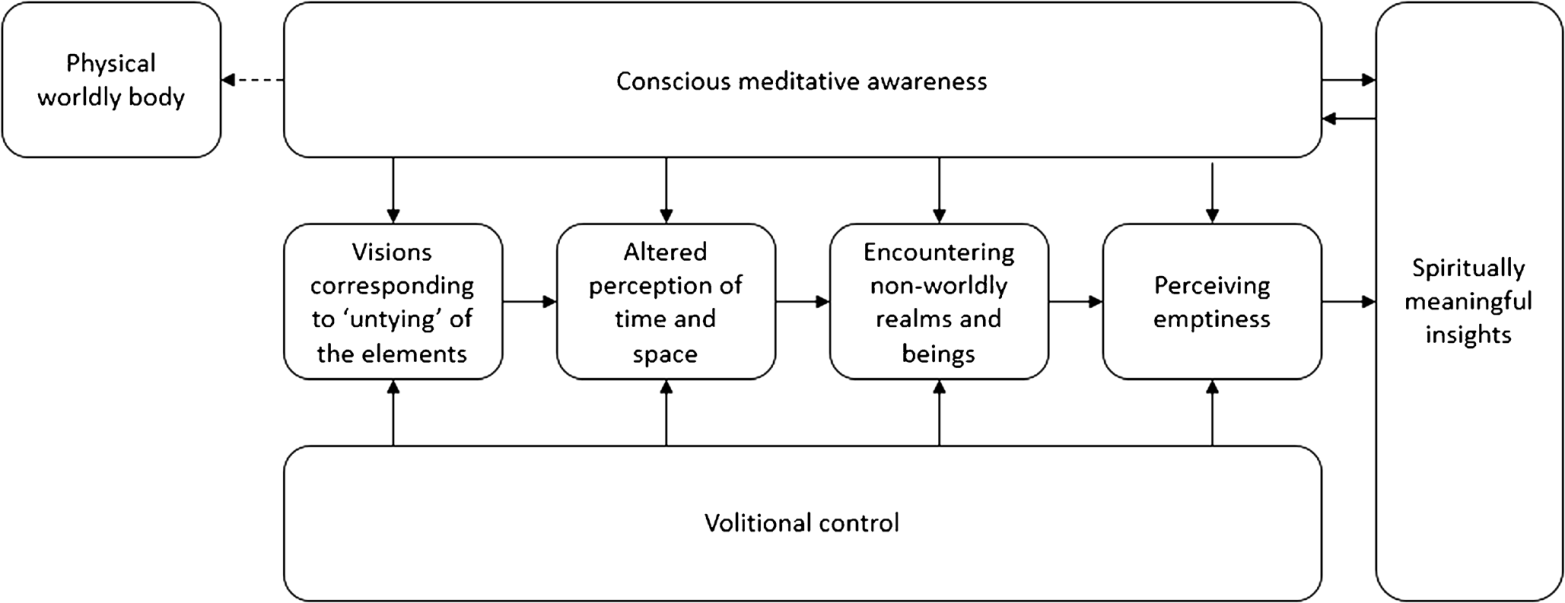
Working model showing the interaction of MI-NDE content, cognitive and meta-cognitive processes and concepts

The present sample of advanced Buddhist meditators derived spiritually significant insights from engaging in the practice of the MI-NDE. In particular, they used the MI-NDE as a means of preparing for death and letting go of attachments to physical worldly existence. The MI-NDE commenced by participants “disconnecting” from their physical worldly body whilst experiencing visions and/or feelings that corresponded to what they deemed to be a process of “untying” from the five bodily/natural elements. Having “let-go” of their body in this manner, participants were able to experience “spaceless space” and “timeless time”. This allowed them to experience visions of what they perceived to be future or past occurrences, and to encounter non-worldly realms and beings. During the MI-NDE, participants were fully cognisant of the fact they were experiencing an NDE and retained a partial awareness of their physical worldly body. Furthermore, participants retained volitional control over the NDE and—to a certain extent—could dictate its content and duration. Of key significance to participants was understanding that the component features of the MI-NDE were “empty” of inherent existence and that although the MI-NDE elicited spiritually meaningful insights, such insights were not to be dwelled upon or assigned a “mystical” status. A working model showing the interaction of MI-NDE content, cognitive and meta-cognitive processes and concepts is described in Fig. 4.

## Discussion

The present study employed mixed-methods and recruited advanced Buddhist meditators in order to conduct an initial empirical investigation of MI-NDEs. Compared to two control conditions that involved standard meditations without inducing an NDE, the MI-NDE condition prompted significantly greater pre-post increases in NDE profundity, mystical experiences and non-attachment. Furthermore, participants demonstrated significant increases in NDE profundity across the longitudinal 3-year study period. No significant relationship was observed between NDE profundity and duration of MI-NDE. Findings from the qualitative study demonstrated that participants (i) were consciously aware of experiencing an NDE, (ii) retained volitional control over the content and duration of the MI-NDE and (iii) elicited a rich array of non-worldly encounters and spiritual experiences.

Scores on the NDE Scale for the MI-NDE condition were considerably higher than the cut-off score for an NDE (i.e. ≥ 7; Greyson 1983) and ranged between 24 and 30 (total permissible range = 0–32). In conjunction with the sizeable difference in NDE Scale scores between the MI-NDE and control conditions (range for control meditation conditions = 6–10), this suggests that participants experienced a genuine form of NDE and that outcomes relating to the MI-NDE condition were not simply the product of a standard or profound form of meditation. Thus, it appears that the MI-NDE referred to in ancient Buddhist texts (i) exists as an empirically investigable phenomenon and (ii) is a valid form of NDE according to conventional assessment criteria. Furthermore, the fact that pre-post scores for mysticism and non-attachment were significantly greater for the MI-NDE versus control conditions demonstrates that the MI-NDE was more “spiritually potent” compared to standard meditation practices. This is consistent with findings from studies of “regular” NDEs that have reported significant gains in spiritual insights and awareness following an NDE (Khanna and Greyson 2014).

Given that NDEs (whether regular or meditation-induced) elicit spiritually meaningful insights, it is unsurprising in the present study that NDE profundity increased over time. This outcome is likely to be a function of “practice makes perfect” and participants continuing to refine the meditative skills that they employ whilst engaging in, and directing the content of, NDEs. However, no significant relationship was observed in the present study between NDE profundity and duration of a particular MI-NDE session. This accords with findings of no relationship between length of unconsciousness and perceived length of NDE from studies of medically caused NDEs (Parnia et al. 2007). A plausible explanation is provided in the Buddhist literature where some Buddhist teachers assert that for the spiritually adept (i) a single second can be experienced as a lifetime and (ii) the present moment is continuously changing and never actually crystallises into existence (i.e. it exists only as a relative concept) (Shonin and Van Gordon 2014b). Thus, the absence of a significant relationship between NDE profundity and duration appears to support claims in both the Buddhist and empirical NDE literature that conventional limits of time do not apply during NDEs (Sogyal 1998; van Lommel 2011). This is consistent with the outcomes from the qualitative analysis conducted as part of the present study where participants reported that they experienced “timeless time” during the MI-NDE.

Study outcomes demonstrated both convergent and divergent factors in terms of the degree to which the MI-NDE differs from a regular NDE. Almost all of the 16 items on the NDE Scale received affirmative responses (i.e. 1 = mildly/ambiguously present or 2 = definitely present) by the majority of participants, suggesting that the features referred to in the NDE Scale (e.g. timelessness, OBE, encountering non-worldly realms and beings, feelings of immense peace and being united with the universe, etc.) also arise during an MI-NDE. The only exception to this was Item 2 (Were your thoughts speeded up?) that did not receive a single affirmative response. Furthermore, no participant responded with a score of 2 (i.e. feature definitely present) to Item 16 (Did you come to a border or point of no return?). A feasible explanation for these observations is that in order to reach advanced stages of meditative development, Buddhist meditators have to embrace the principle of “boundlessness” and seek to transcend relative concepts such as time and space (Van Gordon et al. 2016b). Thus, in line with the aforementioned qualitative outcomes where participants reported experiencing “timeless time” and “spaceless space”, it is perhaps understandable why the present sample of advanced meditators did not provide affirmative responses to questions relating to spatial borders and/or the speeding up of psychological processes (i.e. because the concept of “speeding up” becomes redundant in the absence of time).

The qualitative analysis also identified a number of MI-NDE features that have not to date been recognised as a common feature of a regular NDE. More specifically, it appears that the MI-NDE can be distinguished from the NDE due to the fact (i) it can be consciously induced; (ii) participants retain volitional control over the content and duration of the MI-NDE; (iii) in the early stages of the MI-NDE, there manifests a phase—involving a sequence of visions and sensations—that participants attribute to becoming “untied” from the natural elements; (iv) participants retain a partial awareness over their worldly physical body; (v) participants experience scenes from past lifetimes (i.e. rather than only their current life); and (vi) participants perceive the content of the MI-NDE to be of the nature of “emptiness” and, in some cases, an expression or creation of their own mind.

None of the abovementioned MI-NDE features contradict or are incompatible with the established features of a regular NDE. Rather, we would argue that they add novel dimensions to the NDE and that their occurrence can be attributed to the fact that participants—all of whom were advanced Buddhist meditators—have dedicated decades of their lives to accruing meditative experience (as previously reported, on average, participants had practiced meditation for nearly 30 years and engaged in the MI-NDE practice three times per year). In other words, cultivating and refining the meditative and metacognitive skills to induce, and retain volitional control over NDEs, is inevitably going to afford individuals greater possibilities in terms of the range of NDE features they can experience. Furthermore, unlike regular NDEs where individuals are generally unconscious and only become aware of the fact they have experienced an NDE after the event (van Lommel 2011), participants in the present study reported that they retained meditative awareness throughout the entire NDE episode. Therefore, the participants’ recollection of the MI-NDE—that in many cases was captured within 24 h of the MI-NDE concluding—is likely to be less subject to recall bias.

### Limitations

Although findings from the present study appear to extend the component features of the NDE, they should be considered in light of their limitations. Of particular note is that some participant scores on the NDE Scale for the two control meditation conditions slightly exceeded the threshold (i.e. ≥ 7) for an NDE (standard meditation = 7.17 [SD = 1.34], standard meditation with death contemplation = 8.0 [SD = 1.28]). One explanation for this could be that participants were not familiar with the NDE experience at the high end of the continuum, and thus over-rated the intensity of their experience. Furthermore, as previously discussed, a small number of items on the NDE Scale did not receive a single affirmative response and, based on outcomes elicited during the qualitative analysis, appeared to be incongruous with some core Buddhist meditative principles. In the absence of a scale specifically designed to assess MI-NDEs (i.e. because until now the content of an MI-NDE has not been empirically defined), the NDE Scale was administered in the present study as it was deemed to have sufficient face validity to serve as a rudimentary assessment for an MI-NDE. However, the scale may not have adequate specificity in terms of differentiating between experiences arising during meditation versus those arising during NDEs. Furthermore, it may not accurately assess the profundity or full range of experiences that can arise during an MI-NDE. Additional study limitations were the fact that (i) participant experiences were exclusively assessed using self-report measures, (ii) the sample size was understandably small (i.e. because very few individuals possess the meditative experience necessary to induce an NDE during meditation) and (iii) the sample exclusively comprised Buddhist meditation practitioners meaning that interpretations of the MI-NDE arising due to religious predispositions were not controlled for.

The present study appears to confirm the existence of the MI-NDE referred to in ancient Buddhist texts. Findings demonstrate that some advanced Buddhist meditation practitioners use the MI-NDE to foster insight into death-related processes as well as the nature of self and reality more generally. In addition to adding empirical support in terms of the established features of a regular NDE, findings indicate that there are potentially other interpretations of, as well as other dimensions to, NDEs. Whilst the MI-NDE appears to be a valid form of NDE according to conventional assessment criteria, further research is required to replicate the present findings as well as advance understanding of the features that distinguish an MI-NDE from a regular NDE. A key implication is that the present study indicates it would be feasible—including ethically feasible—for future research to recruit advanced meditators in order to assess real-time changes in neurological activity during NDEs. To date, the health risks and ethical challenges associated with conducting such a study in human participants (i.e. as they experience a regular NDE) have rendered this possibility untenable.
